# A Tale of Two LADs (Left Anterior Descending Arteries): Type IV LAD as a Rare Coronary Anomaly

**DOI:** 10.7759/cureus.106528

**Published:** 2026-04-06

**Authors:** Prashant Thandi, Ritesh Pandey

**Affiliations:** 1 Cardiology, King George's Medical College, Lucknow, IND; 2 Cardiology, Chandan Hospital, Lucknow, IND

**Keywords:** coronary angiography, coronary anomalies, dual lad, left anterior descending artery, nste-acs, ste-acs

## Abstract

A dual left anterior descending (LAD) artery is angiographically defined as the presence of two distinct coronary arteries supplying the LAD territory. It represents a rare coronary artery anomaly in the general population but may have important implications during the evaluation and management of acute coronary syndromes (ACS), percutaneous coronary intervention (PCI), and coronary artery bypass grafting (CABG). Accurate recognition of this variant is essential to avoid misinterpretation of coronary angiograms, incomplete revascularisation, and procedural complications. The objective of this case report is to describe the clinical presentation, angiographic characteristics, and management of two patients diagnosed with type IV dual LAD anomaly in the setting of ACS, one presenting with ST-elevation anterior wall myocardial infarction (STEMI) and the other with non-ST-elevation myocardial infarction (NSTEMI). The report also aims to highlight key diagnostic clues and practical considerations for interventional cardiologists and cardiac surgeons when encountering this rare anomaly.

## Introduction

Type IV dual left anterior descending (LAD) artery is an uncommon congenital coronary anomaly, observed in approximately 0.64-1.3% of coronary angiograms [1]. In this variant, a short LAD (s-LAD) originates from the left coronary system and terminates prematurely, whereas a second, elongated LAD arises abnormally from the right coronary artery (RCA). Although generally regarded as benign, it is essential to accurately recognise this anomaly, as an s-LAD can mimic total occlusion, thereby increasing the risk of misdiagnosis. Precise identification is particularly important for the management of acute coronary syndrome and for planning interventions such as coronary artery bypass grafting or percutaneous coronary intervention (PCI). A comprehensive understanding of this anatomical variation is vital to prevent diagnostic errors and to enhance patient care.

The Spindola-Franco classification scheme delineates four dual LAD types based on the origin and course of the s-LAD and long LAD (l-LAD) segments [2]. In Type IV, the s-LAD arises from the left coronary artery, whereas the l-LAD originates anomalously from the right coronary artery and subsequently traverses the anterior interventricular sulcus (AIS) [3]. This classification remains instrumental in identifying dual LAD anomalies. In this anatomical variant, the s-LAD supplies the upper septum and anterior wall, while the l-LAD originates from the RCA and follows the AIS to supply the distal anterior wall and apex [4]. Diagnosis is most commonly established using techniques such as multidetector computed tomography (MDCT) or coronary angiography.

In some cases, the l-LAD might follow a harmful interarterial path between the aorta and pulmonary artery, increasing the risk of symptoms or sudden cardiac death. Therefore, advanced imaging techniques are essential to accurately map the vessel’s course [5]. As noted earlier, careful anatomical evaluation is critical for sound clinical decisions and better patient outcomes, especially in cases of dual LAD anomalies.

## Case presentation

Case 1

A 40-year-old gentleman reported to the cardiology emergency with the primary complaint of acute chest pain for the last 10 hours, accompanied by recurrent episodes of chest pain at rest since then. He has a history of chronic smoking and moderate alcohol consumption. He was non-hypertensive and non-diabetic, exhibiting no family history of coronary artery disease (CAD). The electrocardiogram (ECG) demonstrated ST elevation in the precordial leads (V1-V4).

Two-dimensional echocardiography (2D ECHO) indicated hypokinesia in the LAD territory, with a left ventricular ejection fraction (LVEF) of 45% and mild mitral regurgitation. His hsTrop I was significantly elevated at 3882 ng/L, while the other blood parameters were within normal limits.

**Figure 1:**
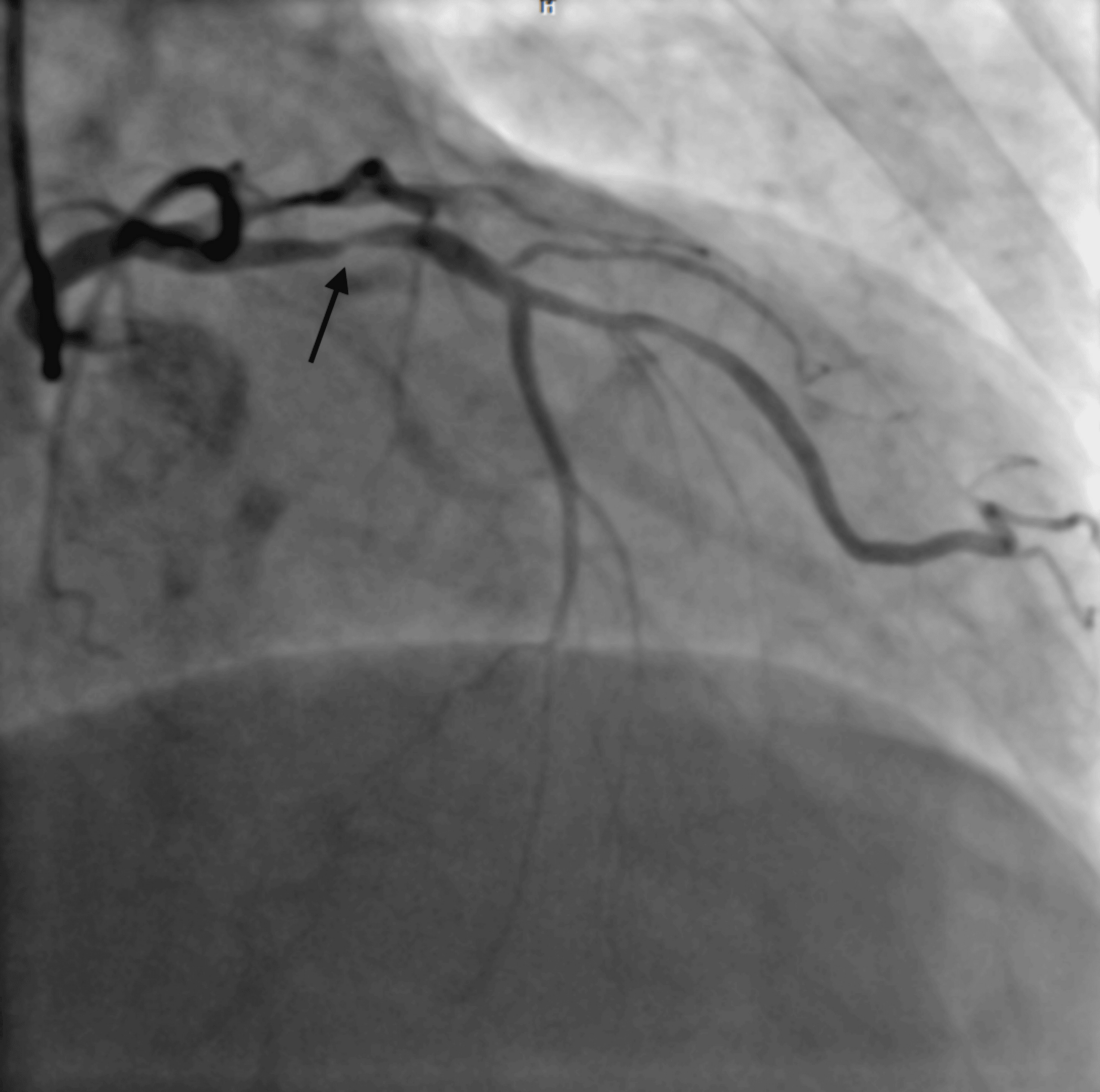
RAO cranial view showing proximal tubular severe disease in LAD with termination into diagonal and septal branches, giving an impression of complete occlusion in the mid-segment of LAD. RAO: right anterior oblique; LAD: left anterior descending artery

**Figure 2:**
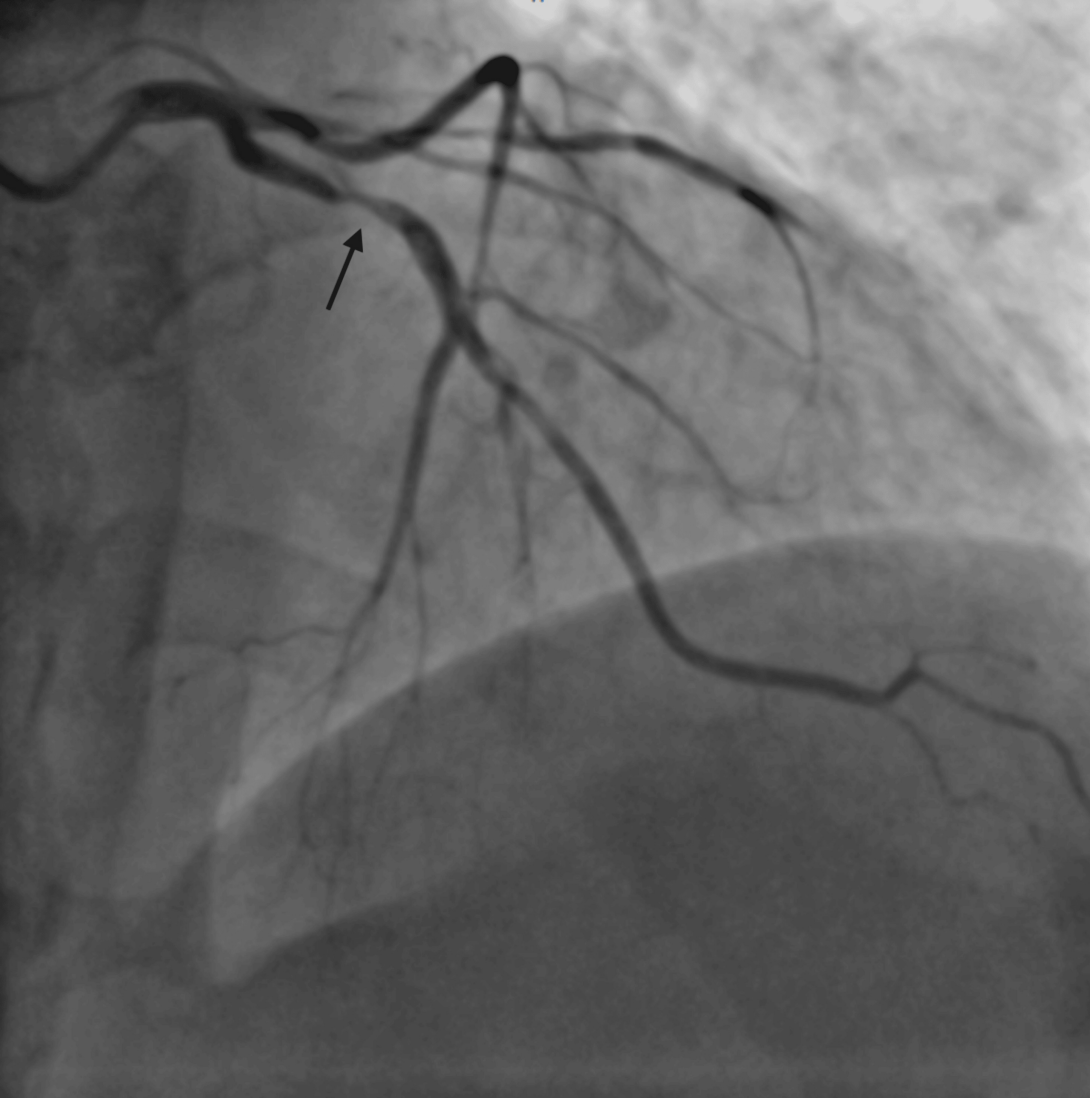
AP cranial view showing proximal tubular severe disease in LAD with termination into diagonal and septal branches. AP: anteroposterior; LAD: left anterior descending artery

**Figure 3:**
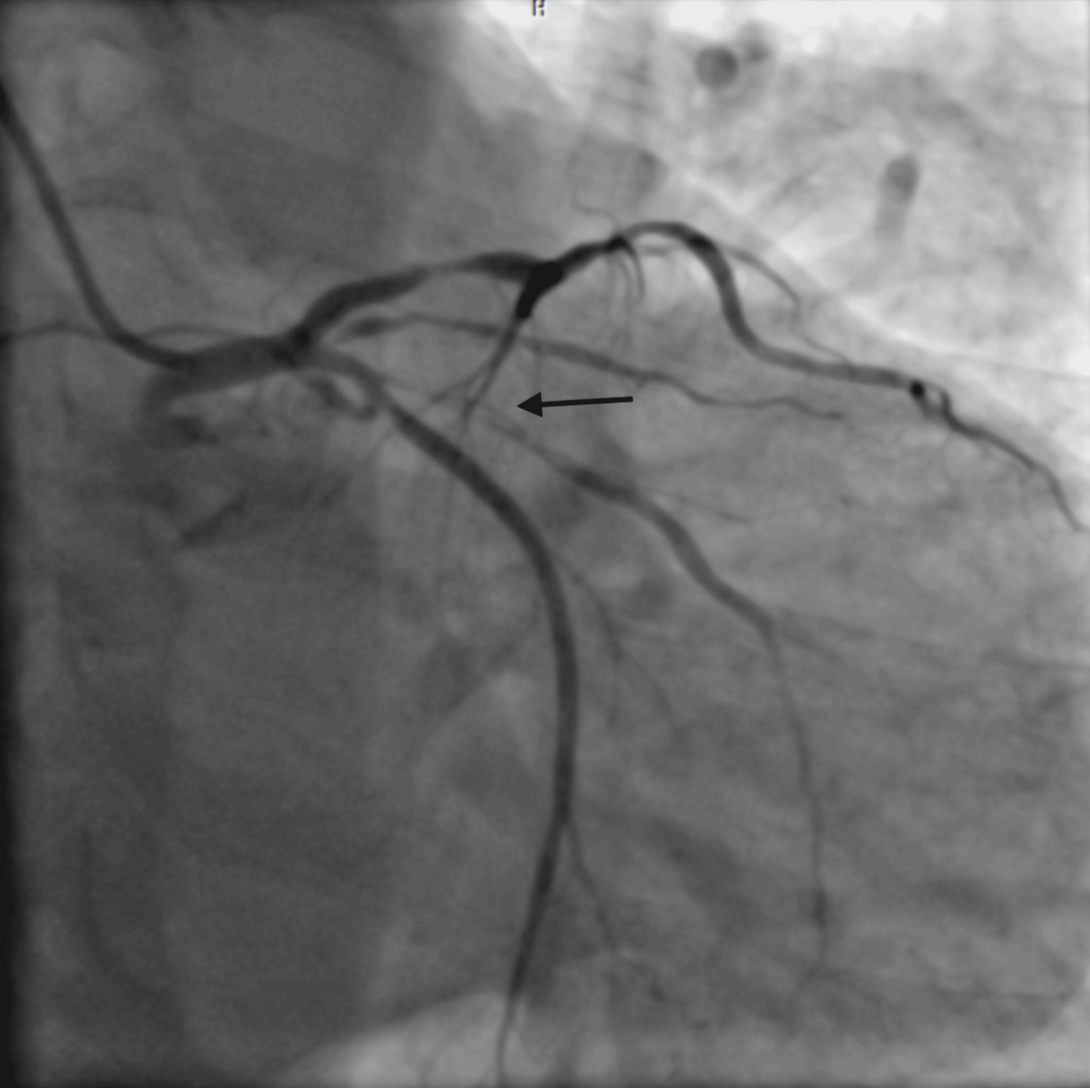
AP-caudal view showing dissection in the OM branch. AP: anteroposterior; OM: obtuse marginal

In view of anterior wall STEMI with ongoing chest pain, the patient was taken up for urgent coronary angiography and primary PCI. Imaging of the left coronary artery demonstrated that the LAD artery originated from the left main coronary artery. This vessel, identified as the s-LAD, had severe tubular disease in its proximal segment and ended prematurely at a high level within the anterior interventricular groove (AIV) (Figures 1, 2). This early termination mimicked mid-LAD occlusion. The initial diagonal and septal regions received branches from s-LAD at its termination. The non-dominant left circumflex artery (LCX) appeared normal, whereas its major obtuse marginal (OM) branch demonstrated severe tubular stenosis with dissection (Figure 3).

**Figure 4:**
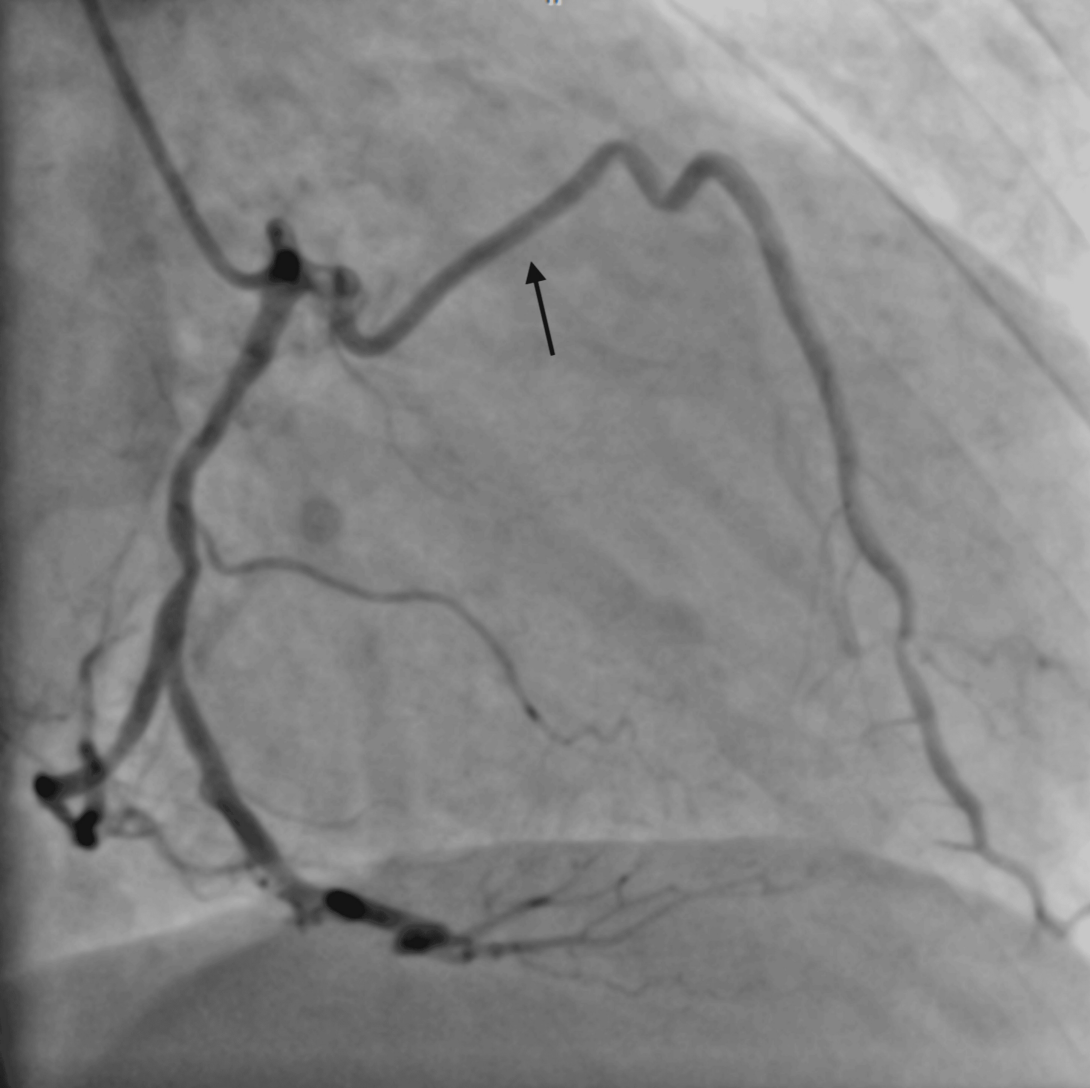
RAO view showing the anomalous origin of the long LAD from the right coronary cusp, separate from the RCA, and its anterior course. RAO: right anterior oblique; LAD: left anterior descending artery; RCA: right coronary artery

**Figure 5:**
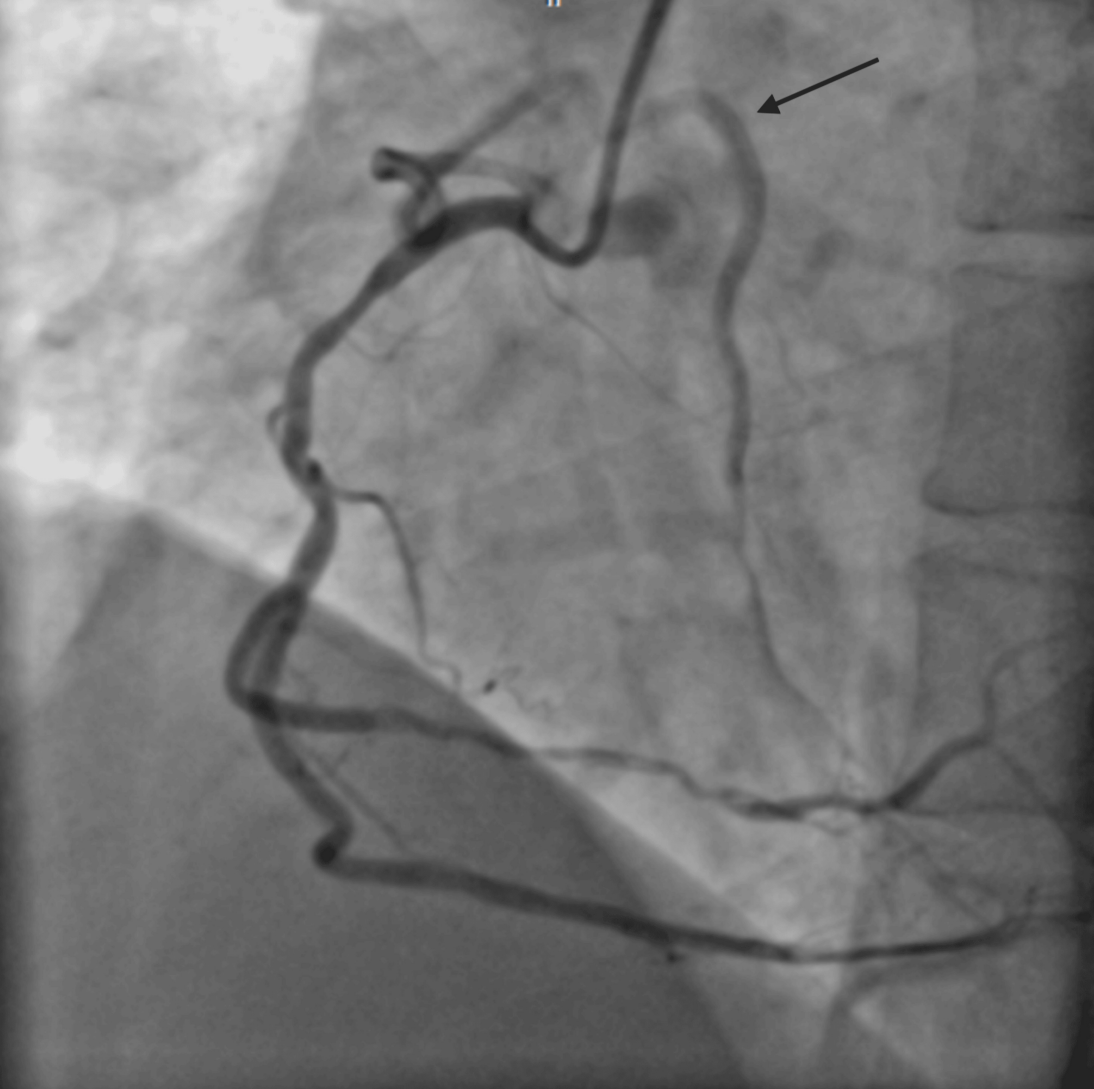
LAO view showing the anomalous origin of the long LAD from the right coronary cusp, separate from the RCA, and its cranial course. LAO: left anterior oblique; LAD: left anterior descending artery; RCA: right coronary artery

The predominant RCA exhibited significant tubular stenosis in both its proximal and distal segments. Angiographic images were obtained in left anterior oblique (LAO) and right anterior oblique (RAO) views to investigate the potential anomalous origin of the LAD artery. The l-LAD arose independently from the right coronary sinus. Its trajectory appeared anterior in the RAO view and cranial in the LAO view, which suggested a course along the right anterior free wall (Figures 4, 5). These findings were consistent with Type IV dual LAD as per the Spindola‑Franco classification.

**Figure 6:**
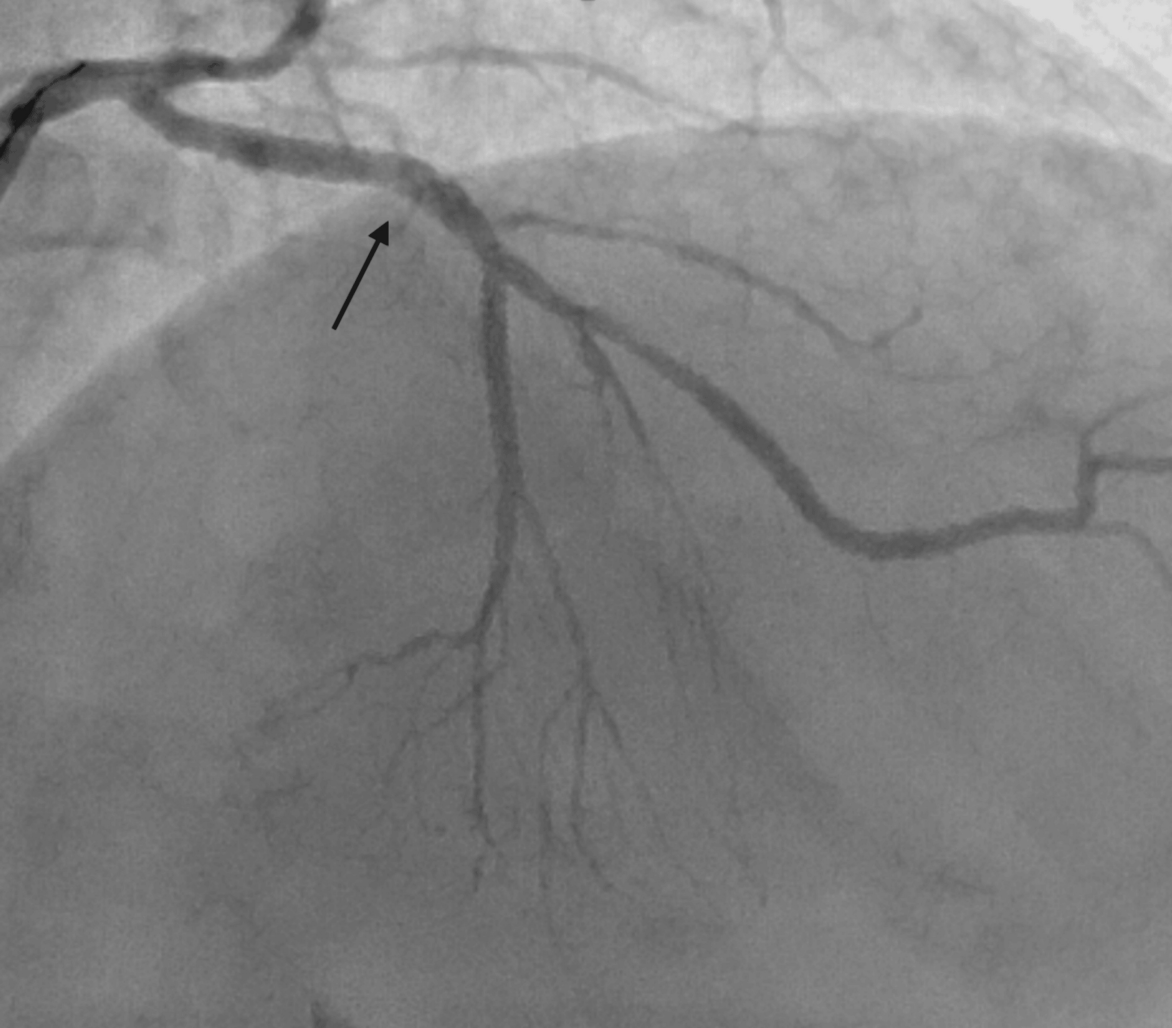
RAO cranial view showing s-LAD post-PCI with DES. RAO: right anterior oblique; s-LAD: short left anterior descending artery; PCI: percutaneous coronary intervention; DES: drug-eluting stents

The patient was advised to undergo percutaneous transluminal coronary angioplasty (PTCA) targeting the s-LAD and RCA, in addition to medical management for OM. PCI with drug-eluting stents (DES) was successfully performed on the s-LAD (one DES) and RCA (staged PCI with one DES after six weeks for complete revascularisation), culminating in a favourable outcome (Figure 6).

Case 2

A 61-year-old female patient who had experienced an acute onset of chest pain a week prior arrived at the cardiology outpatient department. The patient has had hypertension and type 2 diabetes mellitus for 20 years. Her electrocardiogram showed T-wave inversion in leads V1-V4. In addition to minor aortic and mitral regurgitation, the 2D echocardiogram demonstrated mild global left ventricular hypokinesia with an LVEF of 48%. Her high-sensitivity troponin I level was elevated at 750 ng/L. Other blood parameters were within normal limits. The patient was scheduled for coronary angiography.

**Figure 7:**
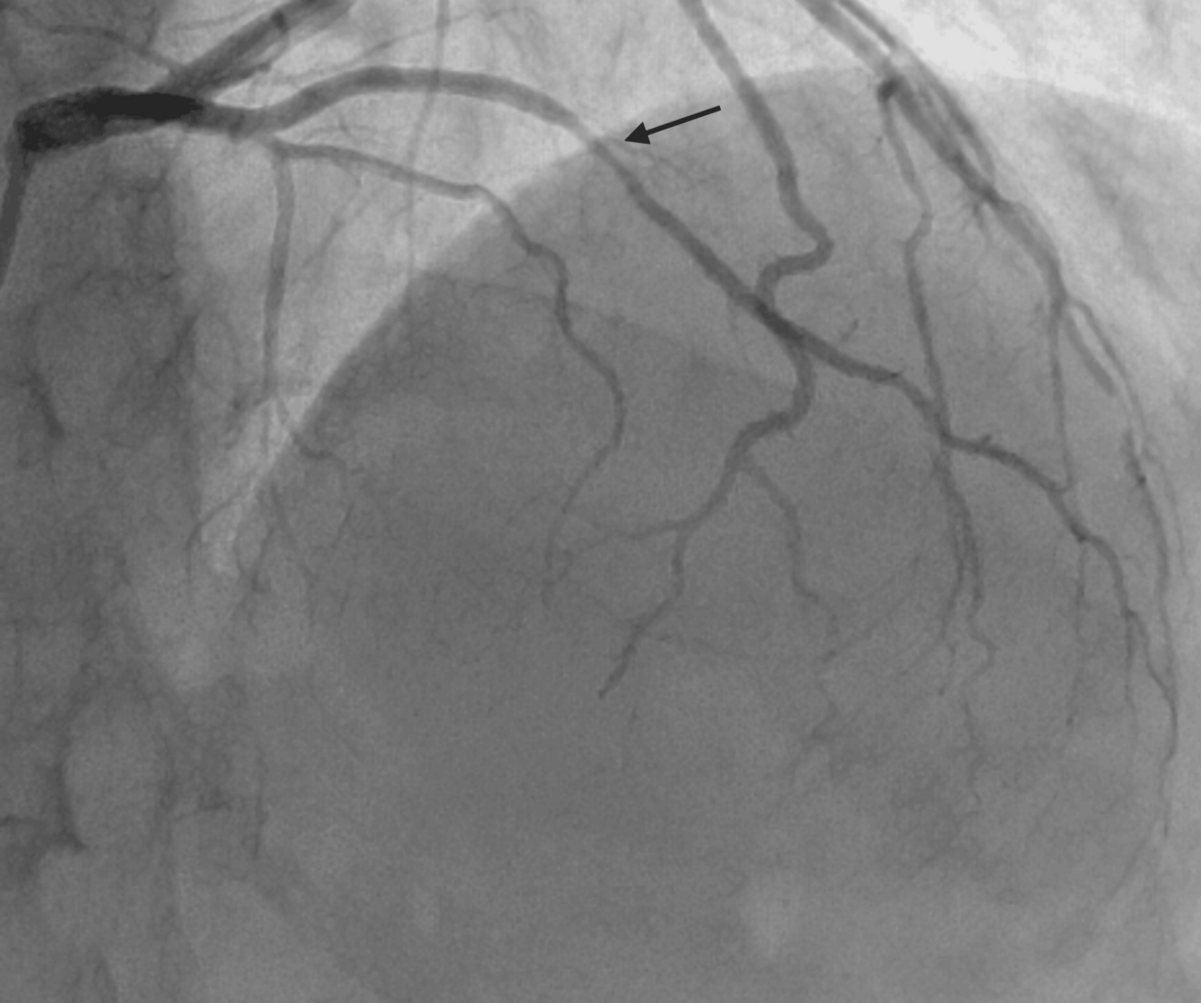
RAO cranial view showing short LAD terminating early and tubular severe disease in the diagonal branch. RAO: right anterior oblique; LAD: left anterior descending artery

The LAD artery arose from the left main coronary artery, as shown on the left coronary angiogram. This vessel, referred to as the s-LAD, appeared normal in course but ended early at a high level within the anterior interventricular groove (AIV). The diagonal branch was a well-sized vessel with significant tubular severe disease (Figure 7). The non-dominant left circumflex artery (LCX) and the obtuse marginal 1 (OM-1) branch were normal. However, OM-2 exhibited a distinct 50% stenosis at its ostium.

**Figure 8:**
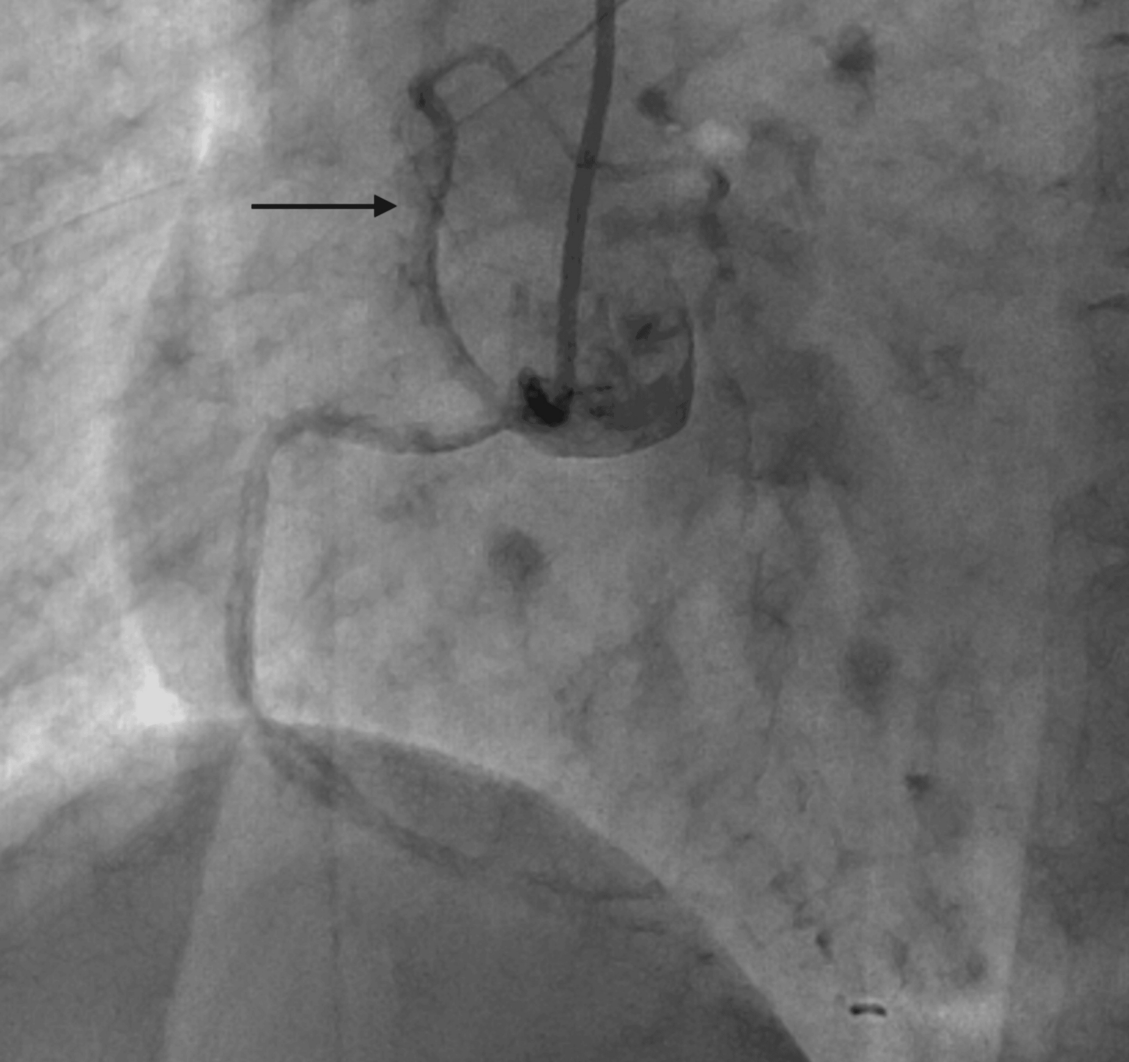
LAO view showing the origin of l-LAD from the right coronary cusp. LAO: left anterior oblique; l-LAD: long left anterior descending artery

**Figure 9:**
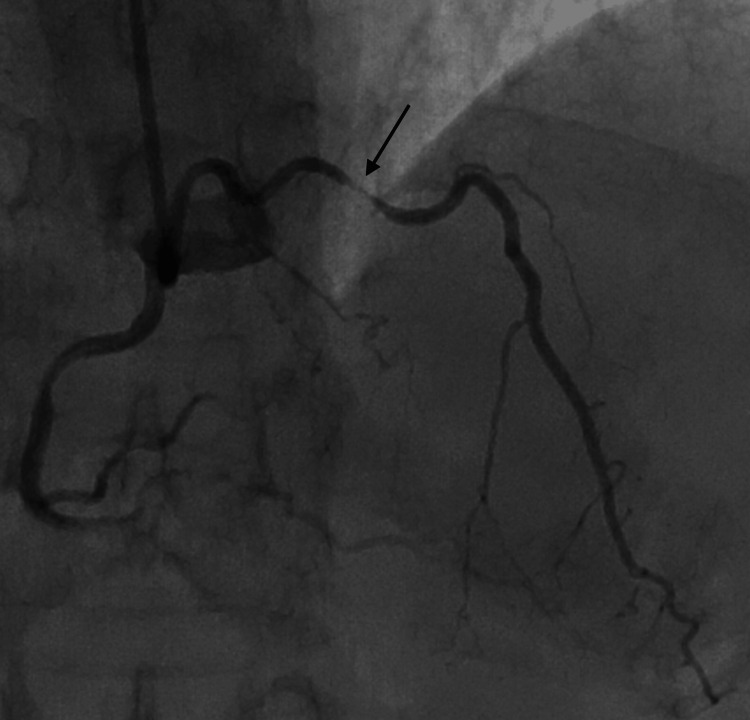
AP cranial view showing the origin of the l-LAD from the right coronary sinus and its anterior course with tubular severe disease. AP: anteroposterior; l-LAD: long left anterior descending artery

The dominant RCA displayed severe tubular stenosis in its distal segment. Angiograms were obtained in RAO, LAO, and anterior cranial (AP cranial) views to assess for an anomalous origin of the LAD artery. The l-LAD emerges from the right coronary sinus. It traversed anteriorly in the RAO view and cranially in the LAO view, indicative of a right anterior free wall trajectory (CT angiography is required to delineate the anomalous course more definitively). The l-LAD exhibited severe tubular disease (Figures 8, 9).

**Figure 10:**
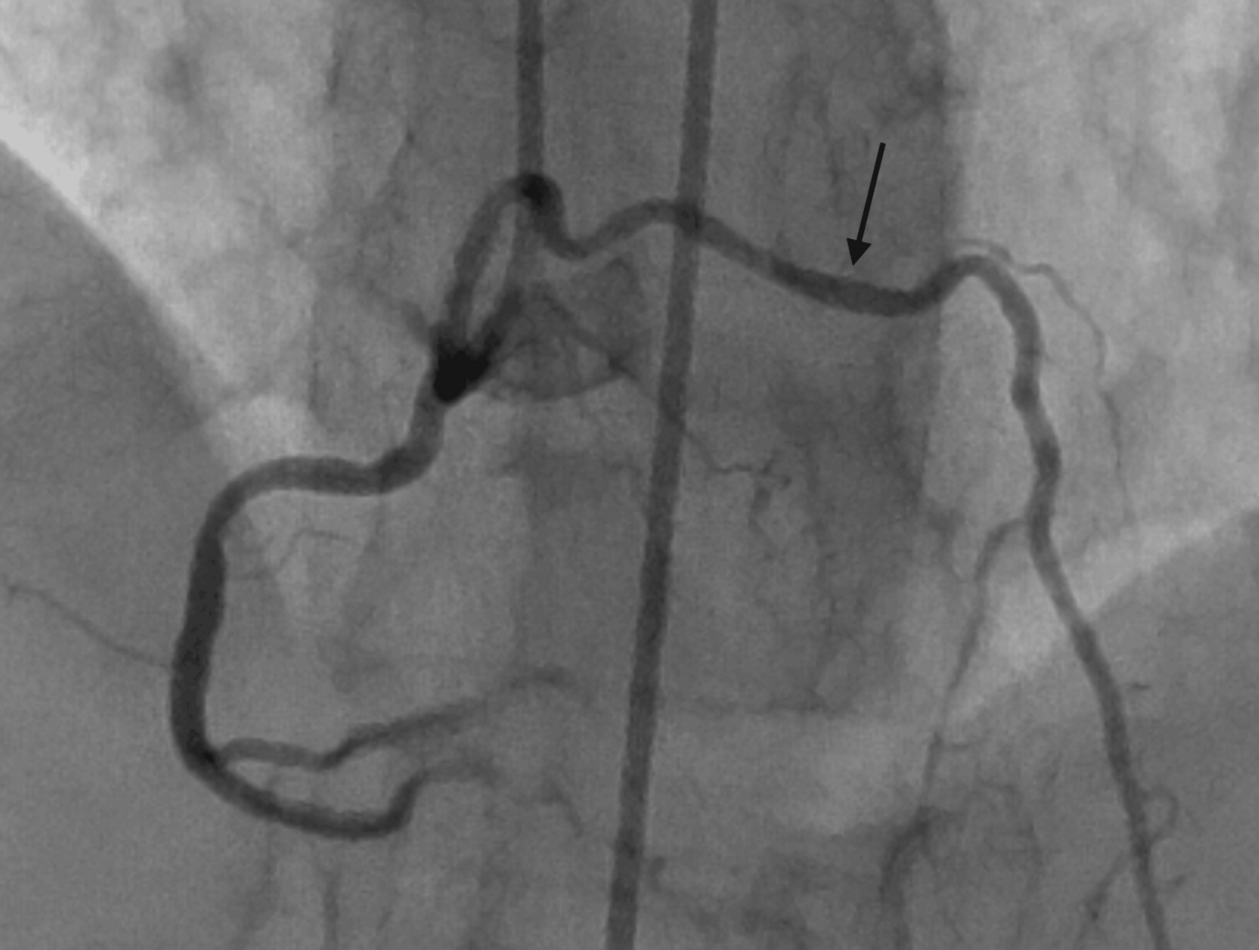
LAO cranial view of l-LAD post-PCI with DES. LAO: left anterior oblique; l-LAD: long left anterior descending artery; PCI: percutaneous coronary intervention; DES: drug-eluting stents

**Figure 11:**
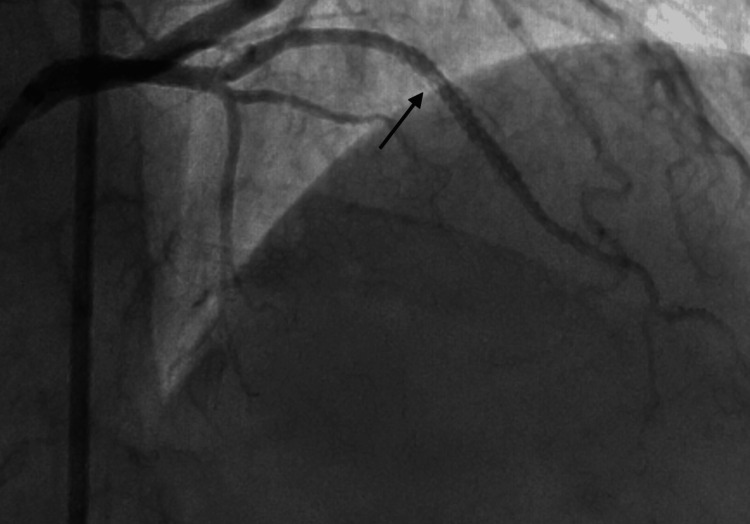
AP cranial view of diagonal branch post-PCI with DES. AP: anteroposterior; PCI: percutaneous coronary intervention; DES: drug-eluting stents

Along with medical management for OM-2, the patient was counselled on PTCA to the diagonal branch, RCA, and the l-LAD. PCI utilising one DES was successfully performed on the l-LAD, RCA, and diagonal branch, resulting in a favourable outcome (Figures 10, 11).

## Discussion

Coronary artery anomalies constitute a group of congenital disorders characterised by diverse manifestations and underlying pathophysiological mechanisms. The prevalence of these anomalies has been reported to range from 0.6% to 1.3% in various angiographic series and approximately 0.3% in autopsy series [6-10]. These anomalies are classified based on course, abnormal origin, and distribution of the affected coronary artery. It is noteworthy that such anomalies are more frequently observed in the RCA than in the left. The incidence of a dual LAD artery is reported to be approximately 1.3% [7]. This anatomical variant consists of two components: an s-LAD and an l-LAD [7,11-13]. The s-LAD usually emerges from the left main coronary artery and ends early at a relatively proximal level within the interventricular groove. The distal part of the interventricular groove is where the l-LAD returns after traversing around the short segment. The l-LAD has a variable course. It courses around the short segment and returns to the distal part of the interventricular groove.

Therefore, when encountering a relatively short or hypoplastic LAD artery, the possibility of an anomalous coronary artery or a variation in the normal coronary artery course should be considered. A long, dominant posterior descending branch of the RCA may extend beyond the apex and end within the AIS, course alongside a parallel diagonal branch, or contribute to a dual LAD artery pattern. The septal LAD is misinterpreted for artery occlusion, whereas an l-LAD may be confused with a conus branch of the RCA. Dual LAD should be considered whenever a paucity of vessels is observed in the apical LAD region, along with a small LAD proper, during coronary angiography.

Based on coronary angiography, dual LAD has been divided into subtypes depending on the origins, courses, and terminations of the s- and l-LAD [6]. Six additional subtypes have been recognised based on CT coronary angiography imaging (Table 1) [11,14,15]. CT coronary angiography can better delineate the exact anomalous course and whether it is malignant.

**Table 1:** Types of dual LAD coronary artery—origin and course. LAD: left anterior descending artery; LMCA: left main coronary artery; RCA: right coronary artery; LCS: left coronary sinus; RCS: right coronary sinus; LV: left ventricle; RV: right ventricle; AIS: anterior interventricular septum; RVOT: right ventricular outflow tract; Type 1-4: Spindola-Franco Classification; Type 5-10: CT-based classification Source: [11,14,15]

Type	LAD Origin		Course	
	Short LAD	Long LAD	Short LAD	Long LAD
Type 1	main LAD	main LAD	Proximal AIS	LV side of the proximal AIS, and then along the distal AIS
Type 2	main LAD	main LAD	Proximal AIS	RV side of the proximal AIS and then along the distal AIS
Type 3	main LAD	main LAD	Proximal AIS	Intramyocardial in the septum proximally, and epicardially in the distal AIS
Type 4	LMCA	RCA	Proximal AIS	Prepulmonic anterior to the RVOT and along the distal AIS
Type 5	LCS	RCS	Proximal AIS	Intramyocardial within the septal crest, then epicardially and enters the distal AIS
Type 6	LMCA	RCA	Proximal AIS	Between the RVOT and the root of aorta and then along the distal AIS
Type 7	main LAD	main LAD	Proximal AIS	LV side of the proximal AIS, and then along the distal AIS
Type 8	LMCA	mid RCA	Proximal AIS	Inferior wall of the RV, then around the apex reaching the distal AIS
Type 9	main LAD	main LAD	Mid AIS	LV side of the mid-AIS, then courses along the distal AIS and terminates before reaching the apex
Type 10	LMCA	RCS	Proximal AIS	Pre-pulmonic, anterior to the RVOT, and then along the distal AIS

The coronary artery that emerges from the right coronary cusp can travel inter-arterially (between the aorta and pulmonary artery), retro-aortically, along the anterior free wall, or along the septum. The initial course in the RAO view can be either anterior or posterior, and in the LAO view, it can be either cranial or caudal.

## Conclusions

Recognition and accurate identification of dual LAD artery anomalies, particularly Type IV LAD, are essential for safe and effective coronary interventions. The cases described show that dual LAD may present with acute coronary syndromes and can easily be misdiagnosed during angiography without careful imaging and anatomical awareness. Using multiple imaging modalities improves diagnostic accuracy and guides appropriate management, as seen with the successful interventions in both reported cases. Clinicians should maintain a high index of suspicion for such anomalies in atypical presentations or when vessel anatomy appears unusual. Systematic classification of dual LAD types, based on origin and course, aids therapeutic planning and helps avoid procedural errors. Incorporating this knowledge into clinical practice enhances patient safety and outcomes in interventional cardiology.
